# Language Differences by Race in the Narrative Section of the Emergency Medicine Standardized Letter of Evaluation

**DOI:** 10.5811/westjem.47304

**Published:** 2025-11-19

**Authors:** Symphony Fletcher, Keme Carter, James Ahn, Paul Kukulski

**Affiliations:** * University of Chicago, Pritzker School of Medicine, Chicago, Illinois.; † University of Chicago, Department of Emergency Medicine, Chicago, Illinois

## Abstract

**Introduction:**

Discrimination and bias based on race/ethnicity permeate the medical education system. Racial disparities in assessment measures can ultimately impact applicants’ Match results. Few studies to date have examined the narrative portion of the emergency medicine (EM) Standardized Letter of Evaluation (SLOE) for language differences by race. In this study we aimed to determine whether there were language differences by race in the narrative portion of the EM SLOE.

**Methods:**

This study is an analysis of word category frequencies in the narrative portion of the SLOE for applicants applying to EM residency. The sample was drawn from the students who applied to the study institution in 2022. The narrative portion of the SLOE and other applicant factors were collected from the Electronic Residency Application Service (ERAS) applications and de-identified. We compared the number of SLOEs containing predefined keywords by race using chi2 analysis. Keywords were identified in six thematic word categories: agency; standout traits; ability; grindstone habits; achievement; and compassion. We performed logistic regression to determine whether any differences remained after controlling for other factors in the application.

**Results:**

Of 1,104 applicants to the institution, 2,288 SLOEs with self-identified race/ethnicity were available for analysis. Black and Hispanic applicants had higher proportions of SLOEs that contained a compassion word than White applicants (24.9% and 22.4% vs 16.9%, respectively). This finding persisted after controlling for other factors in the application for Black applicants (odds ratio 1.61, 95% CI 1.1–2.36]). There was no evidence of difference in word use by race across other thematic categories.

**Conclusion:**

We found differences in the proportion of SLOEs containing compassion words in the narrative portion of the EM SLOE between Black and White applicants, with Black applicants being described with compassion language more frequently. However, we found no difference in any other word category, indicating less overall disparity than other narrative assessment studies.

## INTRODUCTION

Discrimination and bias based on race permeate the medical education system. In particular, these factors have been shown to influence subjective and objective assessment measures used for the residency Match.^1^–^4^ Disparities in assessment measures can ultimately impact applicants’ Match results.^5^,^6^ A recent study on the emergency medicine (EM) Standardized Letter of Evaluation (SLOE) demonstrated differences in the rankings on the letter by race, after controlling for other markers of performance.^7^ The EM SLOE has become the component of a residency application that program directors value most when selecting students to interview and rank; therefore, bias within this tool will affect Match outcomes for applicants to EM residency.^8^–^10^ Further, better understanding the strengths and limitations of the EM SLOE will help leaders in other specialties create equitable standardized letters as more specialties adopt them.^11^

In addition to the numerical rankings, the EM SLOE contains a narrative section, in which writers are instructed to provide “detail on strengths, explaining growth opportunities or lower ratings from above, and highlighting anything else you feel like we should know about this student.”^12^ Previous studies on narrative assessment have shown bias in terms of the language used for different groups such as sex or race.^3^,^13^,^14^ One study showed that significant differences exist in the language used to describe White vs Black applicants on the Medical Student Performance Evaluation (MSPE).^3^ Evidence suggests that residency program directors rely on descriptors within narrative summaries to influence their impression of applicants, both negatively and positively.^15^ Studies have also shown that there is bias toward under-represented in medicine (UIM) students in the use of positive attribute language in letters of recommendation for residency.^14^ Consequently, negatively biased narratives could decrease applicants’ Match potential.

Only one single-center study to date has examined the narrative section of the EM SLOE for language differences by race. The authors found that Black and UIM students were more likely to be described with communal language as compared to White and non-UIM students.^16^ On the other hand, two studies examining word differences by sex on the EM SLOE demonstrated that the narrative section was “relatively free of gender bias.”^17^,^18^ Our specific aim in this study was to determine whether there were language differences by race in the narrative section of the EM SLOE.

## METHODS

### Study Design

This study is a cross-sectional, descriptive linguistic analysis of word category frequencies in the narrative section of the SLOE for applicants applying to EM residency.

### Setting and Participants

The study population includes medical students who applied to EM residency during the 2022–2023 application season. The study sample was drawn from the students who applied to the study EM residency program in 2022. Applicant data were downloaded from the Electronic Residency Application Service (ERAS) system for each student included in our study. This study was approved by the institution’s institutional review board (IRB23-0426).

### Study Protocol

The narrative section of the SLOE (with identifiers removed), student race, degree to be earned (MD/DO/IMG), Alpha Omega Alpha Honor Medical Society (AOA) status, and sex were collected from ERAS applications and de-identified. Data collection and de-identification was performed by a member of the research team (PK). Students self-identified their race/ethnicity during ERAS submission, with the following options: “American Indian or Alaska Native,” “Asian,” “Black or African American,” “Hispanic, Latino, or of Spanish origin,” “Native Hawaiian or other Pacific Islander,” “White,” “Other,” or “Unknown.” Students were able to select as many as needed or leave this section blank.

**Population Health Research Capsule**

What do we already know about this issue?

*Narrative bias in medical evaluations can disadvantage under-represented applicants and affect residency Match outcomes.*

What was the research question?

*Are there racial differences in word use within emergency medicine Standardized Letters of Evaluation narratives?*

What was the major finding of the study?

*Black applicants had more compassion language (OR 1.61, 95% CI 1.10–2.36, P<.05) vs White applicants; no other differences were found.*

How does this improve population health?

*Identifying narrative bias in residency evaluations supports equitable selection, improving diversity and long-term health equity.*

We assessed word category frequencies using the Linguistic Inquiry and Word Count (LIWC) application (Pennebaker Conglomerates, Inc, Austin, TX). This linguistic analysis program measures the use of words within text documents based on predefined or individually designed word category dictionaries. This tool has been validated and used in similar prior studies.^17^,^18^ Based on prior research and extensive literature review—including a landmark study on the MSPE using these categories—we examined six thematic categories: standout traits; ability; agency; grindstone habits (ie, work ethic); achievement; and compassion.^3^,^19^,^20^

### Outcomes

We compared word category frequency in the narrative section of the SLOE by race. Our primary outcome was the use of a word from a thematic category at least one time in the narrative section of the SLOE, similar to previous work on the MSPE.^3^ We compared Black, Asian, and Hispanic students to White students, and UIM students, comprising Black, Hispanic, American Indian/Alaska Native, and Native Hawaiian/Pacific Islander students. to non-UIM students.^21^ We built a LIWC word category dictionary using the six non-overlapping word categories. Each category was populated with word options using prior literature and “add dictionary words” function within LIWC (Table 1).^3^

### Analysis

We assessed for differences in frequency of a word in each thematic category appearing at least once in the narrative portion by comparing SLOEs from applicants of each individual race to White applicants’ SLOEs, using chi-squared analysis. (Not included were American Indian/Alaska Native, and Native Hawaiian/Pacific Islander students due to insufficient sample size for statistical analysis.) Chi-squared tests were also used to evaluate whether there was an association between UIM status and proportion of SLOEs with a word category used at least once. We performed a multivariable logistic regression for each word category to demonstrate the effect of race on at least one word appearing in each category after controlling for sex, AOA status, USMLE (US Medical Licensing Examination) Step 1 score, and medical school type (MD, DO, international medical graduate [IMG]). Word category analyses were conducted using Stata v17 2021 (StataCorp LLC, College Station, TX).

## RESULTS

In 2022, 1,186 applicants applied to the institution’s EM program. Of those applicants, 1,117 had at least one SLOE and 1,104 of those applicants had a self-identified race/ethnicity. In total, 2,288 SLOES with applicant self-identified race/ethnicity were available for analysis. The applicants represented in our sample were similar to the national applicant pool with respect to race and UIM status (Table 2). In our study sample, applicants were more likely to identify as female, be applying from US allopathic medical schools, and inducted into the AOA. Applicants were less likely to be applying from US osteopathic medical schools) or IMG programs. Study applicants were 54.5% White, 19.1% Asian, 9.2% Black, 11.1% Hispanic, 1.3% American Indian or Alaska Native, and 0.27% Native Hawaiian or other Pacific Islander.

Compared to SLOEs for White applicants, the narrative section of SLOEs submitted for Black and Hispanic applicants were significantly more likely to contain a compassion word (24.9% and 22.4% vs 16.9%, respectively) (Table 3).

Compared to SLOEs for non-UIM applicants, SLOEs submitted by UIM applicants were significantly more likely to contain a compassion word (23.4% vs 17.4%). There were no other differences in SLOEs containing a word from the study categories by race. After controlling for USMLE Step 1 score, medical school type, AOA membership, and sex via multivariable logistic regression, SLOEs submitted by Black applicants were shown to have a significantly higher use of a word from the “compassion” category, OR 1.61 (95 CI 1.10–2.36). There were no differences by race for any other word category after controlling for the above variables.

Regression models did show that objective measures such as medical school type and Step 1 score were significantly associated with the primary outcome of one word used in each category (Table 4). Specifically, standout traits, ability, grindstone characteristics, compassion, and achievement were significantly associated with one or more objective measures. Additionally, female sex was associated with a higher proportion of SLOEs with standout and ability words.

## DISCUSSION

This study revealed minor differences in word category use by race in the narrative section of the EM SLOE. Hispanic applicants and Black applicants had a higher proportion of SLOEs containing a compassion word than White applicants and, similarly, UIM applicants had a higher proportion of SLOEs containing a “compassion” word than non-UIM applicants. These differences in word use only persisted for Black applicants, as compared to White applicants, after controlling for other applicant factors. Word differences were found in multiple categories by Step 1 score and medical school type. Further, we found differences in word use by sex that mirror those found in previous studies on the narrative section of the EM SLOE.^17^,^18^ These differences demonstrate that while this is a convenience sampling, the study was powered to detect differences.

The difference in use of compassion words that we found is similar to previous work in narrative assessment, which has shown UIM learners and female (as compared to male) learners to be more likely to be described with compassion language.^14^,^23^ Additionally, these findings are similar to a recent study on language differences in the EM SLOE by race, which demonstrated that Black and UIM applicants were more likely to be described with communal language.^15^ While different, communal language and compassion language are similar and represent categories that do not describe achievement or traditional competency. Further, the previous study reported a trend toward a difference between Black and UIM students, as compared to White and non-UIM peers, regarding empathetic language, which our study confirms by demonstrating the difference between Black and White applicants regarding compassion language.

This highlights an important contrast; while compassion is a desirable trait, it risks reinforcing the portrayal of UIM applicants as inherently more empathetic or caring, potentially overshadowing their professional competencies and achievements. Previous work has shown that communal language was inversely related to academic hiring and promotion.^19^ Further, a qualitative study of internal medicine fellowship letters of recommendation demonstrated the potential for compassion language to overshadow or replace competency language.^11^ Therefore, while it is encouraging that we did not find differences in any other word category, the difference in compassion language reveals that the narrative section in the EM SLOE is not fully free of bias and highlights the need for further contextual analysis.

In contrast with prior work on narrative evaluation, we did not find any other differences by race. These results suggest that the EM SLOE narrative, while not free from racial bias, may demonstrate less bias than other assessments, such as the MSPE letter and traditional narrative letters of recommendations.^3^,^14^ While prior studies have evidenced bias across personal attribute- and competency-based word categories, our study did not demonstrate significant bias across competency-based terms. These results are encouraging for other specialties looking to follow the Coalition for Physician Accountability recommendations that all specialties adopt a standardized letter of evaluation for the residency application process and provides further evidence for the universal move from traditional letters of recommendation to SLOEs.^24^ Further, given there may be less negative bias in the narrative section as compared to the numerical rankings, it is possible that program directors can ensure a more equitable decision-making process by incorporating the narrative section into decision-making. This also emphasizes the need to be vigilant for bias of omission vs commission, as highlighting compassion language may be at the expense of other descriptors. As programs and program directors have increased awareness of bias in evaluation, the residency selection process must evolve to appropriately weigh evaluations based on potential for bias.

These results open multiple future directions for further understanding how to mitigate racial bias in narrative assessment. It is possible that the word limit on the SLOE (350 words) lends itself to a more concise narrative that leaves less room for biased language. Another possibility is that the SLOE instructions “to explain the rankings and competency-based assessments above” cause the writers to focus more on competency-based behavior and less on personal attributes (eg, friendly, personable, energetic, quiet) that are more prone to bias.^14^ Further study is needed to elucidate the reasons for the decreased bias as compared to other narrative studies, including perspectives from SLOE authors.

## LIMITATIONS

There are multiple limitations to this study. First, this was not a full textual analysis of the narratives on the SLOE, meaning that although there were few word differences, it is possible that the overall message conveyed in the narrative may still reflect bias. Further, more in-depth study, such as narrative performative analysis, will be needed to ensure that language use in the SLOE’s narrative assessment is equitable. Second, although like previous work on narrative assessment, our study is limited by the finite number of words included in each category. Categories with a more exhaustive number of words included may yield differing results. Third, our study population was drawn from the applicant pool of a single institution, which may limit the generalizability of findings. A multi-institutional study would be beneficial to corroborate these results. Finally, while included in the UIM analysis, American Indian or Alaska Native, Native Hawaiian, and other Pacific Islander applicants were not represented well enough in our sample to be included in the analysis by specific race/ethnicity.

## CONCLUSION

There were differences in the proportion of Standardized Letters of Evaluation containing compassion words in the narrative section of the EM SLOE between Black and White applicants, with Black applicants being described with compassion language more frequently. However, no difference existed in any other word category, indicating less overall disparity than other narrative assessments.

## Figures and Tables

**Table 1:** Words in each thematic category used to analyze the narrative section of the Standardized Letter of Evaluation.

Thematic Category	Individual terms
Agency	ambitious, confident, active, motivated, responsible
Standout Traits	exceptional, best, outstanding, superb, excellent, phenomenal, stellar
Ability	intelligent, bright, talent, brilliant, competent, smart, gifted
Grindstone Habits	organized, hardworking, conscientious, diligent
Achievement	perform, earn, skill, success, progress
Compassion	caring, kind, empathy, compassionate

**Table 2:** Applicant demographics in a study of differences in word use in the narrative section of the Standardized Letter of Evaluation in emergency medicine.

Demographic	No. of Applicants in Sample (N = 1,104)	Percentage of Applicants in Sample	Percentage of Applicants in National Applicant Pool^20^
Sex			
Male	565	51.2	59.5
Female	532	48.2	40.4
Another gender identity	7	0.6	
UIM status			
Non-UIM	813	73.6	73.8
UIM	228	20.7	19.6
Race/ethnicity			
Non-Hispanic White	602	54.5	55.2
Asian	211	19.1	18.7
Black	102	9.2	7.8
Hispanic, Latino, or of Spanish origin	123	11.1	10.7
American Indian or Alaska Native	14	1.3	0.98
Native Hawaiian or other Pacific Islander	3	0.27	0.21
Other	63	5.7	5.03
Type of school			
US MD	824	74.6	47.1
US DO	173	15.7	25.4
IMG	107	9.7	27.4
AOA status			
AOA	105	9.5	3.4
Not AOA	999	90.5	96.6

*AOA*, Alpha Omega Alpha Honor Medical Society; *DO*, doctor of osteopathic medicine; *IMG*, international medical graduate; *MD*, doctor of medicine; *UIM*, under-represented in medicine.

**Table 3:** Standard Letters of Evaluation with at least one word in a thematic category in the narrative, by race.

	White	Black	Asian	Hispanic	Non-UIM^a^	UIM^a^
Total SLOEs	1,305	214	484	286	1,783	522
Agency	361 (27.7%)	62 (28.3%)	130 (27.2%)	73 (25.5%)	489 (27.4%)	140 (26.8%)
Standout Traits	850 (65.1%)	142 (66.7%)	319 (65.9%)	174 (60.8%)	1,164 (65.3%)	332 (63.6%)
Ability	203 (15.6%)	30 (14.1%)	72 (14.9%)	38 (13.3%)	275 (15.4%)	69 (13.2%)
Grindstone Characteristics	242 (18.5%)	36 (16.9%)	95 (19.6%)	56 (19.6%)	337 (18.9%)	95 (18.2%)
Compassion	221 (16.9%)	**53 (24.9%)** *	89 (18.4%)	**64 (22.4%)** **	310 (17.4%)	**122 (23.4%)** ***
Achievement	198 (15.2%)	30 (14.1%)	71 (14.7%)	32 (11.2%)	267 (15.0%)	68 (13.0%)

* Denotes significant difference from White by chi-squared test, P < .01.

** Denotes significant difference from white by chi-squared test, P = .03.

*** Denotes significant difference from non-UIM by chi-squared test, P < .01.

a UIM indicates Black, Hispanic, American Indian or Alaska Native, and Native Hawaiian or other Pacific Islander applicant SLOE, non-UIM indicates non-Hispanic White and Asian applicant SLOEs.

*EM*, emergency medicine; *SLOE*, Standardized Letter of Evaluation; *UIM*, under-represented in medicine.

**Table 4:** Odds ratio for a word in a thematic category appearing at least once, by race,

	Agency	Standout	Ability	Grindstone	Compassion	Achievement
White	Reference	Reference	Reference	Reference	Reference	Reference
Black	1.07 (0.75, 1.53)	1.24 (0.87, 1.74)	0.99 (0.63, 1.58)	0.93 (0.61, 1.41)	**1.61 (1.10, 2.36)**	0.93 (0.59, 1.47)
Asian	0.98 (0.76, 1.26)	1.01 (0.79, 1.29)	0.93 (0.68, 1.29)	1.06 (0.79, 1.41)	0.99 (0.73, 1.35)	1.02 (0.74, 1.39)
Hispanic	1.01 (0.74, 1.38)	0.91 (0.68, 1.22)	0.89 (0.59, 1.34)	1.01 (0.71, 1.44)	1.34 (0.95, 1.90)	0.75 (0.49, 1.14)
USMLE Step 1 score	1.00 (0.99, 1.01)	**1.02 (1.01, 1.03)**	**1.01 (1.00, 1.02)**	1.00 (0.99, 1.01)	1.00 (0.99, 1.01)	0.99 (.99, 1.01)
Sex	1.17 (0.96, 1.43)	**1.43 (1.18, 1.74)**	**1.31 (1.02, 1.69)**	0.94 (0.75, 1.19)	1.18 (0.93, 1.49)	0.83 (0.64, 1.01)
AOA	1.08 (0.75, 1.53)	1.35 (0.95, 1.92)	1.29 (0.88, 1.90)	1.00 (0.69, 1.46)	0.98 (0.67, 1.44)	0.65 (0.41, 1.05)
Medical school type*	1.08 (0.97, 1.21)	**0.77 (0.69, 0.85)**	0.99 (0.86, 1.15)	**0.78 (0.68, 0.89)**	**0.80 (0.69, 0.92)**	**0.81 (0.70, 0.94)**

* Medical school types: US private MD; US public MD; DO; international.

Statistically significant findings are in bold (P < .05).

*AOA*, Alpha Omega Alpha Honor Medical Society; *DO*, doctor of osteopathic medicine; *EM*, emergency medicine; *MD*, doctor of medicine.
